# Supplementary material for: Association of Antibody Responses to Helicobacter pylori Proteins with Colorectal Adenoma and Colorectal Cancer
Source: Pathogens. 2024 Oct 14;13(10):897. doi: 10.3390/pathogens13100897 (PMC11510280; doi:10.3390/pathogens13100897)
Supplement: Supplementary file 1 [file pathogens-13-00897-s001.zip › pathogens-3195804-supplementary.pdf]

Supplementary Materials

Supplementary Table S1: Antigens included in *H. pylori* multiplex serology

Bacterium	Antigen Name	Putative function	Antigen specific cut off (MFI) for IgG	Antigen specific cut off (MFI) for IgA
H. pylori	GroEl	Chaperonin GroEL	500	100
	UreA	Urease alpha subunit	800	600
	Hp0231	Hypothetical protein	750	150
	NapA	Neutrophil-activating protein	500	250
	Hp0305	Hypothetical protein	300	100
	HpaA	Neuraminyllactose binding hemagglutinin homolog	700	100
	CagA	Cytotoxin associated antigen A	4000	2000
	HyuA	Hydantoin utilization protein A	1000	200
	Catalase	Catalase	1000	500
	VacA	Vacuolating cytotoxin A	1200	600
	HcpC	Conserved hypothetical secreted protein	750	150
	Cad	Cynnamyl alcohol dehydrogenase ELI3-2	500	100
	HP1564 (Omp)	Hypothetical protein	1000	350

H. pylori: Helicobacter pylori; MFI: median fluorescence intensity. References: [21]

Supplementary Table S2: Seropositivity to individual *H. pylori* proteins and association with CRC development. Controls were restricted to individuals with no abnormalities detected after colonoscopy.

Secondary antibody	Antigen		NAD (n=37) n (%)	CRC (n=25) n (%)	Adjusted by age and sex OR	95%CI	p-value	q-value
**α-IgA**	>3 proteins	-	33(89)	20(80)				
		+	4(11)	5(20)	0.83	0.56-1.22	0.33	0.33
	GroEl	-	33(90)	19((76)				
		+	4(10)	6(24)	3.09	0.69-13.8	0.13	0.83
	UreA	-	35(95)	23(92)				
		+	2(5)	2(8)	1.42	0.19-10.3	0.72	0.92
	Hp0231	-	34(92)	19(76)				
		+	3(8)	6(24)	2.95	0.62-14.5	0.17	0.83
	NapA	-	36(98)	23(92)				
		+	1(2)	2(8)	3.83	0.28-52.4	0.31	0.87
	HP0305	-	36(98)	100(100)				
		+	1(2)	0(0)				
	HpaA	-	34(92)	24(96)				
		+	3(8)	1(4)	0.43	0.04-4.52	0.48	0.91
	CagA	-	25(68)	17(68)				
		+	12(32)	8(32)	0.96	0.30-3.03	0.95	0.96
	HyuA	-	33(90)	21(84)				
		+	4(10)	4(16)	1.49	0.31-7.16	0.61	0.91
	Catalase	-	29(79)	23(92)				
		+	8(21)	2(8)	0.30	0.05-1.71	0.17	0.83
	VacA	-	32(87)	22(88)				
		+	5(13)	3(12)	0.84	0.17-4.07	0.39	0.91
	HcpC	-	32(87)	24(96)				
		+	5(13)	1(4)	0.28	0.02-2.81	0.28	0.87
	Cad	-	100(100)	23(92)				
		+	0	2(8)				
	HP1564	-	33(90)	22(88)				
		+	4(10)	3(12)	0.96	0.17-5.32	0.96	0.96

**α-IgG**	>3 proteins	-	21(57)	10(40)				
		+	16(43)	15(60)	0.78	0.57-1.04	0.33	0.33
	GroEl	-	19(52)	10(40)				
		+	18(48)	15(60)	2.10	0.67-6.59	0.20	0.57
	UreA	-	30(81)	17(68)				
		+	7(19)	8(32)	2.14	0.58-7.84	0.24	0.57
	Hp0231	-	32(87)	17(68)				
		+	5(13)	8(32)	2.26	0.58-8.73	0.23	0.57
	NapA	-	27(73)	18(64)				
		+	10(27)	9(36)	1.88	0.58-6.06	0.28	0.57
	HP0305	-	29(79)	20(80)				
		+	8(21)	5(20)	1.59	0.39-6.45	0.51	0.62
	HpaA	-	34(92)	22(88)				
		+	3(8)	3(12)	2.80	0.43-17.9	0.27	0.57
	CagA	-	20(55)	12(48)				
		+	17(45)	13(52)	1.48	0.49-4.44	0.47	0.62
	HyuA	-	31(84)	15(60)				
		+	6(16)	10(40)	3.02	0.87-10.4	0.08	0.57
	Catalase	-	31(84)	22(88)				
		+	6(16)	3(12)	0.92	0.19-4.43	0.92	0.92
	VacA	-	33(90)	23(92)				
		+	4(10)	2(8)	0.70	0.12-4.14	0.70	0.80
	HcpC	-	32(87)	18(72)				
		+	5(13)	7(28)	2.51	0.67-9.44	0.17	0.57
	Cad	-	35(95)	22(88)				
		+	2(5)	3(12)	3.03	0.43-21.0	0.26	0.57
	HP1564	-	20(55)	12(48)				
		+	17(45)	13(52)	1.73	0.56-5.37	0.33	0.59

logistic regression analysis adjusted by age and sex. q-value: p-value adjusted after False Discovery Rate. Ab: antibody; OR: Odd Ratio; CI: Confidence Interval; NAD: no abnormalities detected; *H. pylori*: *Helicobacter pylori*; +: Positive; -: Negative; Statistically significant p and q-values are indicated in bold.

Supplementary Table S3: Seropositivity to individual *H. pylori* proteins and association with advanced adenoma development. Controls were restricted to individuals with no abnormalities detected after colonoscopy.

Secondary antibody	Antigen		NAD (n=37) n (%)	AA (n=82) n (%)	Adjusted by age and sex OR	95%CI	p-value	q-value
**α-IgA**	>3 proteins	-	33(89)	69(84)				
		+	4(10)	13(16)	0.82	0.48-1.33	0.42	0.42
	GroEl	-	33(90)	69(84)				
		+	4(10)	13(16)	1.63	0.47-5.63	0.43	0.72
	UreA	-	35(95)	76(92)				
		+	2(5)	6(8)	0.95	0.16-5.46	0.96	0.96
	Hp0231	-	34(92)	77(93)				
		+	3(8)	5(7)	0.58	0.12-2.70	0.49	0.73
	NapA	-	36(98)	73(89)				
		+	1(2)	9(11)	5.29	0.62-44.6	0.12	0.46
	HP0305	-	36(98)	81(98)				
		+	1(2)	1(2)	0.35	0.02-6.08	0.35	0.72
	HpaA	-	34(92)	76(92)				
		+	3(8)	6(8)	0.82	0.18-3.70	0.80	0.88
	CagA	-	25(68)	65(79)				
		+	12(32)	17(21)	0.46	0.18-1.16	0.10	0.46
	HyuA	-	33(90)	72(87)				
		+	4(10)	10(13)	1.15	0.32-4.13	0.82	0.88
	Catalase	-	29(79)	74(90)				
		+	8(21)	8(10)	0.30	0.09-1.00	0.50	0.46
	VacA	-	32(87)	73(89)				
		+	5(13)	9(11)	0.82	0.24-2.75	0.75	0.88
	HcpC	-	32(87)	77(93)				
		+	5(13)	5(7)	0.44	0.11-1.74	0.24	0.61
	Cad	-	100(100)	76(92)				
		+	0	6(8)				
	HP1564	-	33(90)	75(91)				
		+	4(10)	7(9)	0.79	0.20-3.06	0.73	0.88
**α-IgG**	>3 proteins	-	21(57)	30(37)				

	+	16(43)	52(63)	0.78	0.55-1.09	0.30	0.30
GroEl	-	19(52)	37(45)				
	+	18(48)	45(55)	1.06	0.47-2.41	0.87	0.87
UreA	-	30(81)	55(65)				
	+	7(19)	27(35)	2.11	0.79-5.62	0.22	0.76
Hp0231	-	32(87)	67(81)				
	+	5(13)	15(19)	1.25	0.40-3.90	0.69	0.78
NapA	-	27(73)	53(63)				
	+	10(27)	29(37)	1.50	0.62-3.63	0.36	0.76
HP0305	-	29(79)	60(78)				
	+	8(21)	22(27)	1.47	0.56-3.86	0.42	0.76
HpaA	-	34(92)	67(81)				
	+	3(8)	15(19)	3.11	0.81-11.8	0.09	0.76
CagA	-	20(55)	39(47)				
	+	17(45)	43(53)	1.21	0.53-2.74	0.63	0.78
HyuA	-	31(84)	58(70)				
	+	6(16)	24(30)	1.97	0.70-5.51	0.19	0.76
Catalase	-	31(84)	57(69)				
	+	6(16)	25(31)	1.57	0.55-4.52	0.39	0.76
VacA	-	33(90)	78(95)				
	+	4(10)	14(5)	1.65	0.53-5.14	0.38	0.76
HcpC	-	32(87)	65(79)				
	+	5(13)	17(21)	1.36	0.47-3.94	0.56	0.78
Cad	-	35(95)	72(87)				
	+	2(5)	10(13)	2.46	0.50-12.0	0.26	0.76
HP1564	-	20(55)	42(51)				
	+	17(45)	40(49)	1.15	0.51-2.60	0.73	0.78

logistic regression analysis adjusted by age and sex. q-value: p-value adjusted after False Discovery Rate. Ab: antibody; OR: Odd Ratio; CI: Confidence Interval; NAD: no abnormalities detected. *H. pylori*: *Helicobacter pylori*; +: Positive; -: Negative; Statistically significant p and q-values are indicated in bold.

Supplementary Table S4: Seropositivity to individual *H. pylori* proteins and association with polyp development. Controls were restricted to individuals with no abnormalities detected after colonoscopy.

Secondary antibody	Antigen		NAD (n=37) n (%)	Polyp (n=85) n (%)	Adjusted by age and sex OR	95%CI	p-value	q-value
**α-IgA**	>3 proteins	-	33(89)	71(84)				
		+	4(11)	14(16)	0.91	0.23-3.00	0.48	0.48
	GroEl	-	33(90)	73(85)				
		+	4(10)	12(15)	1.71	0.33-4.10	0.80	0.90
	UreA	-	35(95)	78(91)				
		+	2(5)	7(9)	1.19	0.22-6.25	0.83	0.90
	Hp0231	-	34(92)	75(88)				
		+	3(8)	10(12)	1.40	0.35-5.60	0.62	0.90
	NapA	-	36(98)	83(97)				
		+	1(2)	2(3)	1.29	0.10-15.3	0.83	0.90
	HP0305	-	36(98)	100(100)				
		+	1(2)	0(0)				
	HpaA	-	34(92)	79(92)				
		+	3(8)	6(8)	0.71	0.16-3.16	0.65	0.90
	CagA	-	25(68)	58(68)				
		+	12(32)	27(32)	0.93	0.39-2.23	0.88	0.90
	HyuA	-	33(90)	76(89)				
		+	4(10)	9(11)	0.82	0.22-2.99	0.76	0.90
	Catalase	-	29(79)	76(89)				
		+	8(21)	9(11)	0.36	0.12-1.13	0.08	0.56
	VacA	-	32(87)	75(88)				
		+	5(13)	10(12)	0.70	0.21-2.35	0.57	0.90
	HcpC	-	32(87)	75(88)				
		+	5(13)	10(12)	0.70	0.20-2.40	0.57	0.90
	Cad	-	100(100)	84(98)				
		+	0	1(2)				
	HP1564	-	33(90)	80(97)				
		+	4(10)	5(3)	**0.09**	**0.009-0.98**	**0.049**	0.56
**α-IgG**	>3 proteins	-	21(57)	37(44)				

	+	16(43)	48(56)	0.72	0.32-1.62	0.34	0.34
GroEl	-	19(52)	27(31)				
	+	18(48)	58(69)	**2.23**	**0.97-5.12**	**0.05**	0.69
UreA	-	30(81)	55(64)				
	+	7(19)	30(36)	1.61	0.59-4.37	0.34	0.69
HP0231	-	32(87)	67(81)				
	+	5(13)	18(19)	1.11	0.35-3.44	0.85	0.91
NapA	-	27(73)	63(74)				
	+	10(27)	22(26)	0.71	0.27-1.82	0.48	0.69
HP0305	-	29(79)	56(65)				
	+	8(21)	29(35)	1.78	0.70-4.51	0.22	0.69
HpaA	-	34(92)	80(94)				
	+	3(8)	5(6)	0.85	0.18-3.88	0.83	0.91
CagA	-	20(55)	34(40)				
	+	17(45)	51(60)	1.54	0.68-3.48	0.29	0.69
HyuA	-	31(84)	62(75)				
	+	6(16)	23(25)	1.55	0.55-4.38	0.40	0.69
Catalase	-	31(84)	54(63)				
	+	6(16)	31(37)	2.32	0.84-6.39	0.10	0.69
VacA	-	33(90)	72(84)				
	+	4(10)	13(16)	1.19	0.37-3.79	0.76	0.91
HcpC	-	32(87)	68(80)				
	+	5(13)	17(20)	1.22	0.41-3.57	0.71	0.91
Cad	-	35(95)	83(97)				
	+	2(5)	3(3)	0.500	0.07-3.30	0.47	0.69
HP1564	-	20(55)	46(54)				
	+	17(45)	39(46)	0.94	0.42-2.12	0.94	0.94

logistic regression analysis adjusted by age and sex. q-value: p-value adjusted after False Discovery Rate. Ab: antibody; OR: Odd Ratio; CI: Confidence Interval; NAD: no abnormalities detected; *H. pylori: Helicobacter pylori*; +: Positive; -: Negative; Statistically significant p and q-values are indicated in bold.

Supplementary Table S5: Spearman's correlation analysis between *H. pylori* abundance in tissue and antibody response.

Antigen		RQ in colorectal disease tissue[1](n=34)		RQ in normal mucosa(n=29)	
		r*	p-value	r*	p-value
IgA	GroEl	0.09	0.60	0.11	0.56
	UreA	-0.00	0.96	-	-
	HP0231	-0.06	0.71	0.15	0.42
	NapA	-0.00	0.97	-0.02	0.90
	HP305	-	-	-	-
	HpaA	**-0.34**	**0.046**	-0.24	0.19
	CagA	0.00	1.00	-0.18	0.33
	HyuA	0.02	0.87	**0.36**	**0.05**
	Catalase	-0.16	0.35	**0.48**	**0.007**
	VacA	-0.02	0.88	**0.37**	**0.046**
	HcpC	-0.01	0.92	-0.03	0.86
	Cad	-0.28	0.10	0.34	0.07
	HP1564	-0.09	0.60	0.23	0.23
IgG	GroEl	-0.21	0.27	0.22	0.24
	UreA	-0.07	0.69	0.34	0.06
	HP0231	**-0.41**	**0.016**	0.29	0.12
	NapA	-0.30	0.07	0.30	0.11
	HP305	0.19	0.27	0.19	0.30
	HpaA	0.03	0.86	-0.07	0.65
	CagA	-0.13	0.43	0.23	0.22
	HyuA	-0.06	0.73	0.26	0.15
	Catalase	-0.07	0.65	**0.51**	**0.005**
	VacA	-0.09	0.58	**0.37**	**0.048**
	HcpC	-0.28	0.10	-0.02	0.91
	Cad	-0.02	0.87	0.29	0.11
	HP1564	-0.09	0.59	0.24	0.19

Spearman correlation test. *Correlation coefficient. RQ: relative quantification. [1]Colorectal disease tissue includes colorectal cancer and colorectal adenomas; Statistically significant r* and p-values are indicated in bold.
